# Emergency department visit for atrial fibrillation: sex differences in treatment and outcomes in the Global RE-LY AF Registry

**DOI:** 10.1093/eurheartj/ehae319

**Published:** 2024-06-04

**Authors:** Linda S Johnson, Yuxuan Jiang, Judy Luu, Isabelle C Van Gelder, Clare Atzema, David Conen, Marielle Kloosterman, Luciana Armaganijan, Stuart J Connolly, Michael D Ezekowitz, Lars Wallentin, Isabelle Johansson, William F McIntyre, Jonas Oldgren, Jeffrey S Healey

**Affiliations:** Population Health Research Institute, McMaster University, 237 Barton Street East, Hamilton, Ontario L8L 2X2, Canada; Department of Clinical Sciences, Lund University, Jan Waldenströms gata 35, 20502 Malmö, Sweden; Population Health Research Institute, McMaster University, 237 Barton Street East, Hamilton, Ontario L8L 2X2, Canada; Michael G. DeGroote School of Medicine, Faculty of Health Sciences, McMaster University, 90 Main St W, Hamilton, ON L8P 1H6, Canada; Department of Medicine, Division of Cardiology, McGill University Health Centre, 1001 Decarie Boulevard, Montreal, Quebec H4A 3J1, Quebec, Canada; University of Groningen, University Medical Center Groningen, Hanzeplein 1, 9713 GZ, Groningen, The Netherlands; Sunnybrook Health Sciences Centre, Sunnybrook Research Institute, University of Toronto, Canada; Population Health Research Institute, McMaster University, 237 Barton Street East, Hamilton, Ontario L8L 2X2, Canada; Division of Cardiology, Department of Medicine, Faculty of Health Sciences, McMaster University, Canada; University of Groningen, University Medical Center Groningen, Hanzeplein 1, 9713 GZ, Groningen, The Netherlands; Division of Cardiac Arrhythmias and Electrophysiology, Instituto Dante Pazzanese de Cardiologia, São Paulo, Brazil; Population Health Research Institute, McMaster University, 237 Barton Street East, Hamilton, Ontario L8L 2X2, Canada; Sidney Kimmel Medical School and Bryn Mawr and Lankenau Hospitals, Philadelphia, PA, USA; Uppsala Clinical Research Center, Uppsala, Sweden; Population Health Research Institute, McMaster University, 237 Barton Street East, Hamilton, Ontario L8L 2X2, Canada; Population Health Research Institute, McMaster University, 237 Barton Street East, Hamilton, Ontario L8L 2X2, Canada; Uppsala Clinical Research Center, Uppsala, Sweden; Population Health Research Institute, McMaster University, 237 Barton Street East, Hamilton, Ontario L8L 2X2, Canada; Division of Cardiology, Department of Medicine, Faculty of Health Sciences, McMaster University, Canada

**Keywords:** Atrial fibrillation, Sex differences, Females, Stroke/TIA, Emergency department, Population

## Introduction

Studies of sex-based differences in atrial fibrillation (AF) suggest an influence of sex on cardiovascular death and stroke, however, results are conflicting.^1,2^ Discrepant findings could reflect sex-based differences in access to care, but no studies have explored sex-based differences in treatment and outcomes among countries with differing income levels and gender parity. Such data are needed to understand if sex-based care gaps exist and are associated with differences in outcomes. This knowledge could lead to country and sex-specific treatment recommendations.

This study explores how sex differences in AF treatment and outcomes vary between countries based on their economic status and degree of gender parity in the Global RE-LY AF Registry.

## Methods

### Study population and data collection

From the prospective RE-LY registry [*n* = 15 400 patients presenting to an emergency department (ED) with AF in 47 countries between 2007 and 2011],^3,4^ we excluded patients without AF as primary listed reason for ED presentation (*n* = 8561), or missing outcomes or CHADS_2_ score (*n* = 213), resulting in a sample of 6626 patients. We defined rhythm control as treatment with cardioversion, AF ablation, or the use of any anti-arrhythmic drug. Outcomes were obtained at one-year follow-up and included repeated hospitalization for AF, heart failure (HF) hospitalization, stroke or transient ischaemic attack (TIA), and death.^3^

### Statistical analysis

Selected baseline variables are presented as means ± standard deviation, median [interquartile range (IQR)] or proportion. Sex-based differences in treatments and outcomes are presented as crude proportions, with odds ratios (OR) for female sex compared to male sex and *P*-values derived from multi-level logistic regression with a random effect on country, adjusted for CHADS_2_ score, which was the recommended risk stratification tool at the time of study initiation.^4^ To explore the influence on outcome risks by gender-based disparities or economic factors, we stratified on the World Economic Forum (WEF) Global Gender Gap score for 2011, which estimates country-level overall gender parity with a 0–100 score annually. We used World Bank classifications of income for 2011 to group countries as ‘low and lower-middle’, ‘upper-middle’, and ‘high’ income countries. Interaction parameters were assessed in CHADS_2_-adjusted logistic regression models. All statistical analyses were performed using Stata v 17.0 for Mac (StataCorp, College Station, TX, USA). The study conforms to the Declaration of Helsinki and received ethical approval at all sites. All subjects gave written informed consent.

## Results

Overall, females were older (65.5 ± 14.4 vs. 61.5 ± 14.2 years, *P* < .0001), had a higher median CHADS_2_ score [1 (IQR 1) vs. 1 (IQR 2), *P* < .0001], and more permanent AF (21.5% vs. 18.9%, *P* = .008). The ED visit resulted in hospitalization in 56.1% of females and 53.6% of males (*P* = .09).

### Sex-based differences in treatment strategies

Females were less likely to be treated with a rhythm control strategy, receive cardioversion, or AF ablation during follow-up, but were slightly more likely to be treated with anticoagulation (*Table 1*). The effect of female sex on rhythm control therapy utilization differed by country-level gender parity (*P* for interaction .006), and World Bank income groups (*P* for interaction .001) (*Table 1*). Contraindications to anticoagulation were reported in 7.0% of females and 6.0% of males (*P* = .002).

**Table 1:** Population proportion treated with different strategies and proportion of outcomes in males and females, with *P*-values and odds ratios for female sex compared to male sex, derived from CHADS_2_-adjusted multi-level logistic regression models with a random effect for country

	Treatment strategies										Outcomes							
	Rhythm control strategy		Cardioversion in ED or during follow-up		AF ablation		Anticoagulation use at discharge		Referred to specialist		Repeat visit to hospital for AF		Repeat visit to hospital for HF		Stroke/TIA		Death	
Full population																		
	M, %	F, %	M, %	F, %	M, %	F, %	M, %	F, %	M, %	F, %	M, %	F, %	M, %	F, %	M, %	F, %	M, %	F, %
	**59.8**	55.0**	**35.8**	**30.4**	**5.0**	2.9*	**45.3**	**46.2**	84.5	81.0	20.2	19.7	5.8	8.0	**1.9**	**3.4**	4.8	5.4
	OR	95% CI	OR	95% CI	OR	95% CI	OR	95% CI	OR	95% CI	OR	95% CI	OR	95% CI	OR	95% CI	OR	95% CI
	**0.83**	0.75–0.92**	**0.82**	0.73–0.91**	**0.68**	0.52–0.89*	**1.12**	1.01–1.25***	0.91	0.78–1.05	1.00	0.88–1.15	1.19	0.97–1.46	**1.59**	1.15–2.29*	1.02	0.81–1.28
Strata of World Bank Income levels																		
Low and lower-middle income^a^	M, %	F, %	M, %	F, %	M, %	F, %	M, %	F, %	M, %	F, %	M, %	F, %	M, %	F, %	M, %	F, %	M, %	F, %
	**50.3**	42.3**	27.0	24.1	0.2	0	26.9	27.1	67.8	67.0	8.8	9.1	6.4	7.8	1.8	2.1	6.6	6.2
	OR	95% CI	OR	95% CI	OR	95% CI	OR	95% CI	OR	95% CI	OR	95% CI	OR	95% CI	OR	95% CI	OR	95% CI
	**0.69**	0.56–0.84**	0.81	0.64–1.03	NA		1.07	0.86–1.33	1.09	0.87–1.36	0.90	0.61–1.33	1.07	0.68–1.67	0.92	0.43–1.97	0.95	0.64–1.42
Upper-middle income^b^	M, %	F, %	M, %	F, %	M, %	F, %	M, %	F, %	M, %	F, %	M, %	F, %	M, %	F, %	M, %	F, %	M, %	F, %
	59.8	62.6	30.9	31.3	3.6	3.0	**38.8**	**43.5**	83.9	78.7	18.8	21.1	6.1	9.2	**2.7**	**5.1**	5.6	6.6
	OR	95% CI	OR	95% CI	OR	95% CI	OR	95% CI	OR	95% CI	OR	95% CI	OR	95% CI	OR	95% CI	OR	95% CI
	1.12	0.92–1.36	0.99	0.81–1.22	0.83	0.50–1.39	**1.24**	1.01–1.53***	0.83	0.64–1.07	1.01	0.80–1.28	1.39	0.98–1.98	**1.69**	1.03–2.75***	1.21	0.82–1.79
High income^c^	M, %	F, %	M, %	F, %	M, %	F, %	M, %	F, %	M, %	F, %	M, %	F, %	M, %	F, %	M, %	F, %	M, %	F, %
	**64.2**	58.2***	**42.8**	**34.1**	**8.0**	4.9***	57.6	62.1	92.8	92.8	26.4	26.3	5.4	7.1	**1.4**	2.9***	3.6	4.0
	OR	95% CI	OR	95% CI	OR	95% CI	OR	95% CI	OR	95% CI	OR	95% CI	OR	95% CI	OR	95% CI	OR	95% CI
	**0.83**	0.71–0.97***	**0.76**	0.65–0.89*	**0.66**	0.48–0.91*	1.07	0.91–1.26	0.91	0.68–1.23	1.03	0.87–1.23	1.07	0.79–1.46	**1.94**	**1.15–3.28**	0.90	0.61–1.34
Strata according to World Economic Forum Global Gender Gap scores																		
Q1 (lowest gender parity)^d^	M, %	F, %	M, %	F, %	M, %	F, %	M, %	F, %	M, %	F, %	M, %	F, %	M, %	F, %	M, %	F, %	M, %	F, %
	**46.7**	36.1**	21.5	18.0	0.7	0.1	28.6	29.7	64.6	62.4	5.1	4.2	2.7	3.1	0.8	0.6	6.0	5.2
	OR	95% CI	OR	95% CI	OR	95% CI	OR	95% CI	OR	95% CI	OR	95% CI	OR	95% CI	OR	95% CI	OR	95% CI
	**0.67**	0.54–0.82**	0.82	0.64–1.06	0.25	0.03–2.21	1.07	0.86–1.35	1.02	0.82–1.28	0.88	0.53–1.46	1.19	0.64–2.24	0.81	0.24–2.72	0.90	0.57–1.40
Q2^e^	M, %	F, %	M, %	F, %	M, %	F, %	M, %	F, %	M, %	F, %	M, %	F, %	M, %	F, %	M, %	F, %	M, %	F, %
	60.8	60.7	40.6	36.9	**3.4**	**1.4**	48.1	52.0	**97.7**	95.9***	14.5	17.6	6.3	8.2	2.3	2.8	5.3	5.3
	OR	95% CI	OR	95% CI	OR	95% CI	OR	95% CI	OR	95% CI	OR	95% CI	OR	95% CI	OR	95% CI	OR	95% CI
	0.90	0.72–1.12	0.80	0.65–1.00	**0.40**	0.19–0.84***	1.13	0.90–1.43	**0.53**	0.28–0.97***	1.14	0.86–1.51	1.07	0.75	1.14	0.60–2.15	0.86	0.54–1.35
Q3^f^	M, %	F, %	M, %	F, %	M, %	F, %	M, %	F, %	M, %	F, %	M, %	F, %	M, %	F, %	M, %	F, %	M, %	F, %
	58.5	58.2	40.6	39.5	3.2	2.2	36.6	34.8	78.3	73.2	23.5	27.3	9.5	12.4	**2.8**	**6.5**	6.2	8.0
	OR	95% CI	OR	95% CI	OR	95% CI	OR	95% CI	OR	95% CI	OR	95% CI	OR	95% CI	OR	95% CI	OR	95% CI
	1.07	0.85–1.32	1.04	0.83–1.31	0.71	0.38–1.33	1.19	0.94–1.51	0.91	0.71–1.17	1.15	0.90–1.46	1.07	0.75–1.53	**2.03**	1.19–3.44*	1.21	0.81–1.83
Q4 (highest gender parity)^g^	M, %	F, %	M, %	F, %	M, %	F, %	M, %	F, %	M, %	F, %	M, %	F, %	M, %	F, %	M, %	F, %	M, %	F, %
	69.0	65.9	**38.3**	28.6*	10.6	7.4	60.9	67.2	92.0	93.0	33.0	29.5	4.9	8.3	**1.5**	**3.5**	2.7	3.5
	OR	95% CI	OR	95% CI	OR	95% CI	OR	95% CI	OR	95% CI	OR	95% CI	OR	95% CI	OR	95% CI	OR	95% CI
	0.89	0.72–1.09	**0.74**	0.60–0.91*	0.80	0.57–1.11	1.10	0.90–1.34	1.02	0.70–1.49	0.90	0.73–1.10	1.40	0.96–2.06	**2.09**	1.12–3.90***	1.12	0.66–1.92

Bold values denote statistical significance.

AF, atrial fibrillation; ED, emergency department; HF, heart failure; TIA, transient ischaemic attack; Q, quartile.

^a^Consists of Ukraine, Nigeria, Tanzania, Cameroon, Kenya, Mozambique, Uganda, Senegal, Sudan, and India, and includes 1727 individuals and 155 repeat visits to hospital for AF, 123 hospitalizations for HF, 34 stroke events, and 110 deaths.

^b^Consists of Argentina, Brazil, Ecuador, Chile, Colombia, Venezuela, Russia, Latvia, Turkey, Iran, South Africa, Thailand, and China, and includes 1925 individuals and 384 repeat visits to hospital for AF, 148 hospitalizations for HF, 76 stroke events, and 117 deaths.

^c^Includes Canada, USA, Denmark, Sweden, Ireland, the UK, Austria, Germany, the Netherlands, Italy, Spain, Australia, Bulgaria, the Czech Republic, Hungary, Poland, Slovakia, the United Arab Emirates, Saudi Arabia, Singapore, Japan, and Korea, and includes 2974 individuals and 785 repeat visits to hospital for AF, 182 hospitalizations for HF, 61 stroke events, and 111 deaths.

^d^Q1 consists of Turkey, Saudi Arabia, Iran, Nigeria, Cameroon, India, and Korea. Includes 1550 individuals and 72 AF hospitalizations, 45 HF hospitalizations, 11 stroke events, and 86 deaths.

^e^Q2 consists of Brazil, Colombia, Venezuela, Italy, the Czech Republic, Hungary, Slovakia, Ukraine, Kenya, Senegal, Japan, and the United Arab Emirates. Includes 1589 individuals and 252 AF hospitalizations, 114 HF hospitalizations, 40 stroke events, and 84 deaths.

^f^Q3 consists of China, Thailand, Singapore, Uganda, Mozambique, Tanzania, Russia, Poland, Bulgaria, Australia, Austria, Chile, Ecuador, and Argentina. Includes 1544 individuals and 392 AF hospitalizations, 169 HF hospitalizations, 72 stroke events, and 109 deaths.

^g^Q4 consists of Canada, USA, Denmark, Ireland, Sweden, the UK, Germany, Holland, Spain, Latvia, and South Africa. Includes 1924 individuals and 605 AF hospitalizations, 123 HF hospitalizations, 46 stroke events, and 59 deaths.

*P < .01; **P < .001; ***P < .05.

### Sex-specific differences in one-year AF outcomes

After adjustment for the CHADS_2_ score, female sex was associated with increased risk of stroke [OR 1.59, 95% confidence interval (CI) 1.15–2.19, *P* = .005], which remained significant after adjustment for anticoagulation use (OR 1.60, 95% CI 1.16–2.21, *P* = .004), and among patients with non-valvular AF (*n* = 5209) (OR 1.62, 95% CI 1.15–2.30, *P* = .006), including when using Bonferroni correction in the overall population. This risk was numerically more prominent in upper-middle and high-income countries (*P* for interaction .51), and in the top two quartiles of Global Gender Gap score (*P* for interaction .17) (*Table 1*).

We also tested an extensively adjusted model including age, tobacco use, AF type, alcohol use, anticoagulation use, history of sleep apnoea, HF, diabetes, hypertension, stroke, chronic obstructive pulmonary disease, rheumatic heart disease, myocardial infarction, and severe valvular disease. Results were attenuated, but remained significant (OR 1.42, 95% CI 1.01–1.99, *P* = .045). Female sex was not associated with rehospitalization for AF, hospitalization for HF, or death.

## Discussion

In this global, ED-based registry of AF, females were less likely to be managed with rhythm control, particularly in lower income countries and those with less gender equity. There was no overall sex-related difference in the one-year rate of AF or HF hospitalization or death, but females did have a greater risk of stroke, even after extensive multivariable adjustment.

Studies of sex-based differences in stroke risk in AF need to be interpreted in the context of age, comorbidities, and oral anticoagulation use, and also consider competing risks of mortality affecting males. Increased stroke risk in AF among females has been reported, notably in the ATRIA study,^2^ and an Ontario population-based study.^5^ Other studies, including the PREFER-AF,^1^ which had high anticoagulation rates in both sexes, and a Quebec registry, that used time-updated covariates,^6^ did not detect such a difference. Our analysis adds to the understanding of the increased risk of stroke among females by showing that this is present primarily in countries with high gender parity and in high-income countries.

In contrast to the effect of female sex on stroke, the lower use of rhythm control among females in this study was independent of comorbidities, and most pronounced in countries with lower gender parity. Prior to enrolment into this registry, data from the RACE and AFFIRM studies did not show an advantage for rhythm control.^7,8^ However, recent data from the EARLY-AF and EAST-AFNET4 studies suggest a benefit of rhythm control for prevention of AF recurrence and outcomes.^9,10^ In light of this, it is important that females are offered rhythm control therapies to the same extent as males.

## Conclusions

In the year after an ED visit for AF, females were less likely to receive rhythm control therapy, most notably in lower income countries or those with less gender parity. Despite this, there was no effect of female sex on AF or HF hospitalization or mortality. Female sex was associated with an increased risk of stroke/TIA, even after adjustment for comorbidities and anticoagulant use.

## Declarations

### Disclosure of Interest

L.S.J. receives consulting fees from MEDICALgorithmics. J.S.H. has research grants and speaking fees from BMS/Pfizer, Boehringer-Ingelheim, Boston Scientific, Novartis, Medtronic, and Servier. D.C. has received consultation fees from Roche Diagnostics and Trimedics, as well as speaker fees from Servier and BMS/Pfizer.

## Data Availability

Data will be made available by reasonable request to the corresponding author.

## Pre-registered Clinical Trial Number

None supplied.

## References

[1] Schnabel RB, Pecen L, Ojeda FM, Lucerna N, Rzayeva N, Blankenberg S, et al Gender differences in clinical presentation and 1-year outcomes in atrial fibrillation. Heart 2017;103:1024–30. 10.1136/heartjnl-2016-310406 28228467 PMC5529986

[2] Fang MC, Singer DE, Chang Y, Hylek EM, Henault LE, Jensvold N, et al Gender differences in the risk of ischemic stroke and peripheral embolism in atrial fibrillation: the AnTicoagulation and Risk factors In Atrial fibrillation (ATRIA) study. Circulation 2005;112:1687–91. 10.1161/CIRCULATIONAHA.105.553438 16157766 PMC3522521

[3] Healey JS, Oldgren J, Ezekowitz M, Zhu J, Pais P, Wang J, et al Occurrence of death and stroke in patients in 47 countries 1 year after presenting with atrial fibrillation: a cohort study. Lancet 2016;388:1161–9. 10.1016/S0140-6736(16)30968-0 27515684

[4] Oldgren J, Healey JS, Ezekowitz M, Commerford P, Avezum A, Pais P, et al Variations in cause and management of atrial fibrillation in a prospective registry of 15,400 emergency department patients in 46 countries: the RE-LY Atrial Fibrillation Registry. Circulation 2014;129:1568–76. 10.1161/CIRCULATIONAHA.113.005451 24463370

[5] Buhari H, Fang J, Han L, Austin PC, Dorian P, Jackevicius CA, et al Stroke risk in women with atrial fibrillation. Eur Heart J 2024;45:104–13. 10.1093/eurheartj/ehad508 37647629 PMC10771362

[6] Renoux C, Coulombe J, Suissa S. Revisiting sex differences in outcomes in non-valvular atrial fibrillation: a population-based cohort study. Eur Heart J 2017;38:1473–9. 10.1093/eurheartj/ehw613 28073863

[7] Wyse DG, Waldo AL, DiMarco JP, Domanski MJ, Rosenberg Y, Schron EB, et al A comparison of rate control and rhythm control in patients with atrial fibrillation. N Engl J Med 2002;347:1825–33. 10.1056/NEJMoa021328 12466506

[8] Van Gelder IC, Hagens VE, Bosker HA, Kingma JH, Kamp O, Said SA, et al A comparison of rate control and rhythm control in patients with recurrent persistent atrial fibrillation. N Engl J Med 2002;347:1834–40. 10.1056/NEJMoa021375 12466507

[9] Andrade JG, Wells GA, Deyell MW, Bennet M, Essebag M, Champagne J, et al Cryoablation or drug therapy for initial treatment of atrial fibrillation. N Engl J Med 2021;384:305–15. 10.1056/NEJMoa2029980 33197159

[10] Kirchhof P, Camm AJ, Goette A, Brandes A, Eckardt L, Elvan A, et al Early rhythm-control therapy in patients with atrial fibrillation. N Engl J Med 2020;383:1305–16. 10.1056/NEJMoa2019422 32865375

